# Yarning about Diet: The Applicability of Dietary Assessment Methods in Aboriginal and Torres Strait Islander Australians—A Scoping Review

**DOI:** 10.3390/nu15030787

**Published:** 2023-02-03

**Authors:** Alyse Davies, Julieann Coombes, Jessica Wallace, Kimberly Glover, Bobby Porykali, Margaret Allman-Farinelli, Trinda Kunzli-Rix, Anna Rangan

**Affiliations:** 1 Discipline of Nutrition and Dietetics, Susan Wakil School of Nursing and Midwifery, Faculty of Medicine and Health, The University of Sydney, Sydney, NSW 2006, Australia; 2 Charles Perkins Centre, The University of Sydney, Sydney, NSW 2006, Australia; 3 Aboriginal and Torres Strait Islander Health Program, George Institute for Global Health, Sydney, NSW 2042, Australia; 4 Faculty of Medicine, University of New South Wales, Sydney, NSW 2052, Australia; 5 Boab Health Services, Broome, WA 6725, Australia

**Keywords:** Aboriginal, Torres Strait Islander, dietary intake, dietary assessment

## Abstract

Conventional dietary assessment methods are based predominately on Western models which lack Aboriginal and Torres Strait Islander knowledges, methodologies, and social and cultural contextualisation. This review considered dietary assessment methods used with Aboriginal and Torres Strait Islander populations and assessed their applicability. Four electronic databases and grey literature were searched with no time limit applied to the results. Screening, data extraction and quality appraisal were undertaken independently by two reviewers. Out of 22 studies, 20 were conducted in rural/remote settings, one in an urban setting, and one at the national population level. The most frequently used and applicable dietary assessment method involved store data. Weighed food records and food frequency questionnaires had low applicability. Modifications of conventional methods were commonly used to adapt to Indigenous practices, but few studies incorporated Indigenous research methodologies such as yarning. This highlights an opportunity for further investigation to validate the accuracy of methods that incorporate qualitative yarning-based approaches, or other Indigenous research methodologies, into quantitative data collection. The importance of developing validated dietary assessment methods that are appropriate for this population cannot be understated considering the high susceptibility to nutrition-related health conditions such as malnutrition, overweight or obesity, diabetes, and cardiovascular disease.

## 1. Introduction

Dietary intake is assessed using dietary assessment methods. These methods provide a systematic approach to collect, classify, and synthesise important and relevant habitual food and nutrient intake for an individual or group, which is needed to identify susceptibility to nutrition-related problems such as malnutrition, overweight or obesity, diabetes, and cardiovascular disease, and their causes. Conventional dietary assessment methods are based predominately on Western models which lack Aboriginal and Torres Strait Islander knowledges, research methodologies, and social and cultural contextualisation. Aboriginal and Torres Strait Islander knowledge of food systems, food procurement, and nutrition have always existed as part of the connection to Country and a greater way of knowing (epistemology), being (ontology), and doing (axiology), which encompasses all aspects of life [1,2,3,4]. 

Colonisation has impacted connection to Country through the removal of custodianship of Aboriginal and Torres Strait Islander people’s respective lands [5] and continues to impact Aboriginal and Torres Strait Islander people and communities today in 2023. Forced removal from family, and from Country, prevented access to traditional foods. Under the banner of ‘protection,’ Aboriginal and Torres Strait Islander people were forced onto missions and reserves dictating total dependency on the state. Food rationing, imposed as a means of control, consisted largely of flour, refined sugar, salted meat, and tea, leading to severe malnutrition and reduced immunity [5]. This westernised diet high in sugar, salt, and saturated fat remains problematic for Aboriginal and Torres Strait Islander people today and contributes to the higher rates of nutrition-related conditions such as overweight or obesity, diabetes, and cardiovascular disease. The use of dietary assessment methods, such as the 24-h recall used in the National Aboriginal and Torres Strait Islander Nutrition and Physical Activity Survey 2012–2013, provide population-level data that confirm the extent of these dietary implications of colonisation [6]. 

Scientific research rooted in colonial epistemology [7] of empiricism and rationalism identifies itself as the centre of legitimate knowledge [8], which has resulted in the oppression of Aboriginal and Torres Strait Islander people and continues to prevent better health outcomes [9]. Research which is underpinned by Aboriginal and Torres Strait Islander ways of knowing, being, and doing offers a way to decolonise this space by examining and critiquing the authority of Western research models to reflect Aboriginal and Torres Strait Islander worldviews [10]. Situated within the dominant biomedical/biopsychosocial approach to health and related policy in Australia, dietary assessment methods are used to quantitatively assess dietary intake, consumption patterns, and associations between diet and health outcomes, and to evaluate the success of dietary interventions and health-promotion programs [4,11,12]. 

General limitations of dietary assessment methods are well established, such as validity being influenced by under-reporting or bias in self-report methods such as 24-h recalls [13]. Additional considerations regarding the quality of quantitative data obtained arise when assessing their relevance and applicability for Aboriginal and Torres Strait Islander people and communities. For example, data collected from an urban setting may not be generalisable to remote settings and vice versa, and language barriers may influence the accuracy of responses [13,14]. These dietary assessment methods also did not incorporate Aboriginal and Torres Strait Islander knowledges and methodologies during their development. As a result, their use risks further excluding these knowledges, such as a holistic understanding of health, social or family structures [3,4], and methodologies, such as yarning as a culturally appropriate and legitimate tool for gathering data [15,16]. Thus, the use of these dietary assessment methods contributes to the reinforcement of colonial dominance within the nutrition and dietary intake research area [17]. 

This presents an opportunity for innovative research which incorporates quantitative research approaches (such as dietary assessment methods) but also encompasses Aboriginal and Torres Strait Islander ways of “knowing, being and doing” [17]. In doing so, we can develop dietary assessment methods appropriate for Aboriginal and Torres Strait Islander peoples to better assess dietary intake for the prevention of nutrition-related health problems. As such, the objective of this scoping review is to (1) identify the dietary assessment methods used in dietary intake research in Aboriginal and Torres Strait Islander people and communities and (2) determine how applicable these methods are for use. Throughout this review, the term Aboriginal and Torres Strait Islander people is respectfully used when referring to people who identify as being of Aboriginal, Torres Strait Islander, or Aboriginal and Torres Strait Islander origin.

## 2. Materials and Methods

### 2.1. Protocol and Registration

The protocol for this scoping review was developed and registered on the Open Science Platform, https://osf.io/ja259/, accessed on 9 November 2022. The findings are reported in line with the Joanna Briggs Institute updated methodological guidance for scoping reviews and PRISMA Extension for Scoping Reviews [18,19].

### 2.2. Inclusion Criteria

#### 2.2.1. Participants

Aboriginal and Torres Strait Islander people residing in any region of Australia were included. Studies were excluded if Aboriginal and Torres Strait Islander people did not make up at least 50% of the sample population, if the data was unable to be extracted from non-Aboriginal Australians, or if the sample population included only infants, children, or pregnant or breastfeeding women. 

#### 2.2.2. Concept

Studies that considered dietary intake in Aboriginal and Torres Strait Islander people and/or communities using one, or a combination of, dietary assessment methods were included. Dietary assessment methods included methods such as store data, 24-h recall, food frequency questionnaire (FFQ), weighed food record (WFR), diet history, direct observation, and conversational methods. Yarning is a conversational, informal interview through which participant and interviewer journey together to gather information during the interview process [15]. Supplementary Table S3 provides a brief summary of the main features contained in each dietary assessment method. Studies that utilised only short dietary assessment instruments such as screeners or short questionnaires were excluded.

#### 2.2.3. Context

This review considered studies conducted at an individual, household, and community level from all areas of Australia as well as at the national level. 

### 2.3. Types of Studies

To determine the range and nature of dietary assessment methods used with Aboriginal and Torres Strait Islander people, the eligibility criteria for included study types was broad. All primary study designs, government-produced reports, and other policy documents were included. Systematic reviews, meta-analyses, study protocols, conference abstracts, and secondary analyses of data from primary studies were excluded. Any primary studies with no reference to the dietary assessment method used, which addressed food availability or food security, or studies that only focused on supplements or alcohol intake, were excluded. The reference lists of publications selected for full text screening and relevant, but excluded, systematic reviews were examined for additional eligible papers. The language of studies was restricted to English and there was no set date limit for the year of completion of the studies.

### 2.4. Search Strategy

A search strategy was developed by researchers (A.D., J.C., J.W., K.G.) and an experienced university librarian (M.C.). The search strategy included terms that related to, or described, the diets and dietary assessment of Aboriginal and Torres Strait Islander populations. An electronic literature search was conducted using the following databases: Ovid (MEDLINE, Embase), Scopus, and Global Health. Grey literature sources, including the Australian Indigenous HealthInfoNet, Lowitja, and Google Scholar, were searched. The advanced search function was used for Google Scholar and results were limited to the first 200 records. The search strategy for MEDLINE is shown in Supplementary Table S1, conducted on 31 August 2022.

### 2.5. Selection Process

The identified records from the full search were imported into EndNote 20 (Clarivate Analytics, PA, USA) and duplicates were removed. The citations were then imported into Covidence (Veritas Health Innovation, Melbourne, Australia). The titles and abstracts of the records were screened independently against the inclusion criteria by two non-Aboriginal reviewers (K.G. and J.W.). For those records with potential to be included, the full texts were retrieved. Two reviewers independently screened the full texts against the inclusion and exclusion criteria. The reasons for exclusion after full text screening were catalogued in Covidence. Disagreements regarding inclusion or exclusion of publications were discussed between the two reviewers and, where necessary, resolved through consultation with an Aboriginal researcher (J.C.) and non-Aboriginal researchers (A.D. and A.R.). 

### 2.6. Data Extraction and Charting

Data were extracted and charted using a modified data-charting form based on two existing forms used for the assessment of dietary assessment methods in other populations [20,21]. The information extracted for each included study included the study name, location, date and duration of study, study design, study aims, population, dietary assessment method, specific dietary assessment tools (if identified), origin of the tool, dietary components assessed, nutritional information used, reported strengths and limitations of the method, reported reliability or validity of the method, and reported cultural appropriateness/community involvement. Using Microsoft Excel, two independent non-Aboriginal reviewers (K.G. and J.W.) extracted the data and resolved discrepancies through discussion or consultation with a third non-Aboriginal reviewer (A.D.). Where a study had multiple publications related to it, all publications that satisfied the inclusion criteria were included to allow extraction of all relevant details of the dietary assessment method and its use in the study. Where more than one publication relating to a study was included, references to a ‘study’ in the results refer collectively to all the publications related to it.

### 2.7. Critical Apprasial

To assess the cultural appropriateness and quality of the peer-reviewed papers included in this review, the 2018 South Australian Health and Medical Research Institute (SAHMRI) and the Centre of Research Excellence in Aboriginal Chronic Disease Knowledge Translation and Exchange (CREATE) Aboriginal and Torres Strait Islander Quality Appraisal Tool (QAT) was utilised [22]. Two independent reviewers (K.G. and J.W.) assessed each included peer-reviewed paper against the 14 QAT questions, using the QAT Companion Document as an aid [23]. The appraisal process was supervised by an Aboriginal researcher (J.C.).

Potential responses to the QAT for each appraised paper were ‘yes’, ‘partially’, ‘unclear’, and ‘no’. Responses to each question for each peer-reviewed paper were compiled in a tabular form adapted from an existing format [24]. Papers were assigned a ‘yes’ where they provided clear evidence within the publication for the relevant question. Papers were assigned a ‘partially’ response if some or less detailed evidence was provided for a question. The ‘unclear’ response was used where papers may have addressed the question but did not clearly include either partial or full evidence in the written text. Papers were assigned a ‘no’ if no explicit evidence for the question was reported. Papers that received a ‘yes’ or ‘partially’ answer for at least 10 out of 14 questions were considered to be of high quality and more culturally appropriate [24]. Papers that received a ‘yes’ or ‘partially’ for six to nine questions were considered to be of moderate quality and papers that received a ‘yes’ or ‘partially’ to five or fewer questions were considered to be of low quality and less culturally appropriate [24]. To assess how well each individual question performed across all of the peer-reviewed papers, three marks were assigned for a ‘yes’, two for a ‘partially’, one for an ‘unclear’, and zero for a ‘no’ [25]. 

### 2.8. Synthesis of Results

The results are presented in tabular format with associated narrative summaries which describe how the results relate to the aims of this review. Interpretation of the results sought to consider and value Aboriginal and Torres Strait Islander knowledges around health, nutrition, and gathering of information in conjunction with a western, scientific perspective [4,17]. 

## 3. Results

### 3.1. Search Results

**Figure 1:**
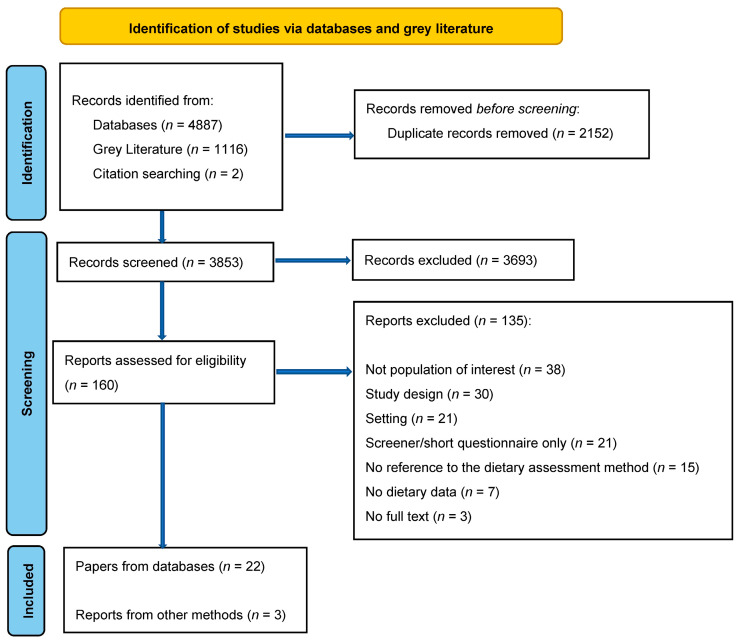
PRISMA flow diagram of record identification and study selection for a scoping review of the applicability of dietary assessment methods in Aboriginal and Torres Strait Islander Australians.

Databases, grey literature, and citation searching identified a total of 6005 records. A total of 2152 duplicates were removed. The titles and abstracts of 3853 records were then screened and 3693 records were excluded. A full text review of 160 records was carried out to assess eligibility and 135 records were excluded. The main reasons for exclusion were a focus on a population that did not fit the inclusion criteria (*n* = 38), an ineligible study design (*n* = 30) or setting (*n* = 21), or the dietary assessment method being a screener or short questionnaire (*n* = 21). Other reasons for exclusion included records which did not contain a reference to dietary assessment methods (*n* = 15), no dietary data (*n* = 7) or no full text (*n* = 3). The PRISMA flow diagram details the selection process (see Figure 1).

### 3.2. Study Selection and Characteristics

Study characteristics are presented in Table 1. In total, 25 publications (peer-reviewed papers (*n* = 22) [26,27,28,29,30,31,32,33,34,35,36,37,38,39,40,41,42,43,44,45,46,47], grey literature reports (*n* = 3) [6,48,49]) from 22 studies were included. The included publications covered a 74-year time period from 1948 to 2022. Of the 22 primary studies, 17 employed one dietary assessment method [6,26,27,28,29,30,31,32,33,34,35,36,37,38,43,44,45,47,49] and five employed two or more dietary assessment methods [39,40,41,42,46,48].

Eight studies took place in the Northern Territory (NT) [28,29,30,31,32,33,34,37,38,45], three in Western Australia (WA) [35,39,40,44] and South Australia (SA) [46,48,49], two in New South Wales (NSW) [42,43], and one in Queensland (QLD) [47] and Victoria (VIC) [36]. One study took place across communities in three states (which were not individually identified) and the NT [27], and one study was Australia-wide [6]. Two studies did not specify their exact locations. One described itself as taking place in a northern coastal and desert community [41] and one in an island community in Northern Australia [26]. 

Twenty studies took place in rural/remote settings [26,27,28,29,30,31,32,33,34,35,36,37,38,39,40,41,42,43,44,45,46,48,49]. One study took place in an urban setting [47] and one in both urban and rural/remote settings [6]. Of the 20 studies from rural/remote settings, 15 assessed community-level dietary intake [26,27,28,29,30,31,32,33,34,35,38,39,40,41,42,45,48,49], four assessed individual-level dietary intake [36,37,43,44] and one assessed household-level dietary intake [46]. The study in an urban setting assessed community-level dietary intake [47]. The study that took place in both urban and rural/remote settings assessed national population-level dietary intake [6]. The size of the studies assessing individual intake varied from 17 to 209 participants [36,37,43,44]. Household intake was assessed for 13 households, each with between four and 13 occupants [46]. The population size of the studies assessing community- or national-level intake varied from 15 to 8515 [28,48]. Population characteristics such as age and gender were not reported in the majority of the included studies as communities wished to remain anonymous.

### 3.3. Dietary Assessment Methods

#### 3.3.1. Store Data

Eleven studies, all in rural/remote settings, utilised store data (either store sales data or store turnover data) as a dietary assessment method [26,27,28,29,30,31,32,33,34,35,41,48,49]. Five studies were located in the NT [28,29,30,31,32,33,34], two in SA [48,49], one in WA [35], one across three unspecified states and the NT [27], and two in unspecified ‘northern’ locations [26,41]. Store sales data were collected from point-of-sale records while store turnover data were collected from store invoices for stock delivered, with both collection methods producing data used to determine community-level dietary quality and intake. A total of nine of the 11 studies utilised store data as a single method to assess community-level intake [26,27,28,29,30,31,32,33,34,35,49], one utilised store data in combination with an adapted 24-h recall [48], and one utilised store data in combination with four other assessment methods (WFR, 24-h recall, FFQ, and diet history) [41]. 

#### 3.3.2. Food Frequency Questionnaires

Five studies (two in NT [37,38], one in VIC [36], one in WA [39,40], and one in a northern coastal and desert community [41]) utilised a FFQ [36,37,38,39,40,41]. Two utilised it as a single method in a rural/remote setting to assess how many times per week individuals consumed a range of everyday foods [36,37] and one utilised it as a single method in a rural/remote setting to assess usual community-level intake based on ration amounts provided [38]. Two studies utilised it in combination with other methods to assess community-level intake in a rural/remote setting [39,40,41]. One of these studies used the FFQ method to assess how frequently foods were consumed within a fortnightly income cycle [41] and the other used a modified FFQ to aid in establishing community-level intake patterns [40]. 

#### 3.3.3. 24-h Recall

Five studies utilised a 24-h recall [6,41,46,47,48]. Two studies used the 24-h recall method as a single method [6,47]. In one study, the method was used in an urban setting to assess changes in community-level intake with a recall conducted at baseline and post–lifestyle intervention [47]. Another study used the method in a national setting to assess population-level intake with two recalls undertaken at least eight days apart for non-remote participants and a single recall for remote participants [6]. Two studies utilised 24-h recall in a rural/remote setting in combination with other methods to assess community-level intake [41,48]. One of these studies conducted a single recall with individual participants [41] while the other conducted multiple adapted recalls with three family groups during each of the four one-month long study periods [48]. Another study utilised a single unstructured, unquantified 24-h recall in combination with observation and conversational methods to assess household-level intake in a remote/rural setting [46]. 

#### 3.3.4. Weighed Food Records

Four studies, all in a remote/rural setting, utilised WFR [41,42,43,44]. The number of days evaluated ranged from a single day [41] to 6 days [43] to 28 days [44]. Two studies used it as a single method to assess individual-level intake [43,44] and two used it in combination with other methods [41,42].

#### 3.3.5. Direct Observation 

Five studies, all in remote/rural settings, utilised observation as a dietary assessment method [40,42,45,46,48]. Four studies used it in assessing community-level intake [40,42,45,48] with the time periods of observation including eight separate days over a two-month period [42], a three-week period [40], four non-consecutive months over a ten- month period [48], and a seven-month study period that included an unspecified time period at each of four settlements [45]. One study used observation, among other methods, to help assess household-level intake with between four and 11 days spent observing each household [46]. 

#### 3.3.6. Conversational Methods

One study utilised a focus group with an open-ended questioning approach after difficulties gathering data with only a quantitative FFQ [39,40]. 

Two studies adopted conversational approaches and referenced ‘yarning’-style methodologies during either the application of their selected methods [46] or as a means of retrospectively assessing the acceptability to participants of their selected method [47].

### 3.4. Reported Validity of Dietary Assessment Methods

Store data was validated by Lee [31] as a method for measuring community-level dietary intake in remote Aboriginal and Torres Strait Islander communities in circumstances where a single community store provided the majority of food supplies between 1989 and 1990. Validation occurred through comparison between store data and trends in biological nutritional status indicators [31]. Another study found higher apparent energy intake based on store data than the individual energy intake recorded from the WFR method, although the majority of nutrients assessed using store data fell within the 95% confidence interval of the WFR method [41]. 

One study measured the relative validity of the 24-h recall method as a method for dietary intake in a remote Aboriginal and Torres Strait Islander community against the WFR method and found poor agreement between the methods for all assessed nutrients [41]. Compared to the WFR method, the 24-h recall method tended to underestimate sugar and complex carbohydrate intake and overestimate intake of protein, fat, and the majority of vitamins and minerals considered [41].

### 3.5. Reported Strengths and Limitations to Assess the Applicability of Dietary Assessments

All strengths and limitations as reported in the publications are shown in Table 2. Studies that utilised 24-h recall [47], a modified food list/questionnaire [38], store data [26,30,35,49], and observation [45,48] reported underreporting of traditional food intake as a limitation. One study reported the ability to adapt a FFQ to include appropriate traditional foods as a strength [39,40].

Studies that utilised 24-h recall [6,41,47] or a FFQ [36,37] reported social desirability bias as a limitation. Store data was reported to not be impacted by this limitation [30,41]. The ability of store data to provide an objective assessment of community-level dietary intake in communities where the local store provides the main source of food was identified as a strength of the method in five studies [27,28,30,31,32,41]. 

FFQs were identified as not being reflective of actual intake or frequency of intake of certain foods, attributed to misunderstandings around concepts of ‘frequency’ and ‘quantity’ [39,40]. The utility of WFRs was identified as being limited due to being incompatible with cultural traditions such as participation in traditional ceremonies and the limited acceptance of the usefulness of recording quantitative measures of intake [41]. 

Studies that used qualitative methods (observation, conversation, focus groups) reported the ability to gather contextual data, for example factors influencing food habits, choices, food distribution within households, and preparation/cooking methods, as a strength [39,40,46,48]. The inability of store data to account for such contextual factors was identified as a limitation of the method [27,30,35,41,48].

### 3.6. Quality Appraisal

The results of the quality appraisal are presented in Supplementary Table S2. A total of six papers were considered ‘high quality’ [28,31,32,33,36,46], two papers ‘moderate quality’ [35,47], and 14 papers ‘low quality’ [26,27,29,30,34,37,38,39,40,41,42,43,44,45]. Two papers, one by Lee 1993 [31] and one by Lee et al. 1994 [32], written about the same study, received the best overall cultural appropriateness score. Of the 22 papers appraised, 19 were published prior to the release of the QAT in 2018 [26,27,28,29,30,31,32,33,34,35,38,39,40,41,42,43,44,45,47]. Four of these papers were considered ‘high quality’ [28,31,32,33] with three written about the same study [31,32,33]. There did not appear to be any discernable trend of improvement in quality over time of papers published up to 2018. Three papers were published after the release of the QAT [36,37,46] and two of these were considered ‘high quality’ [36,46]. 

Inclusion of some level of Indigenous Governance was the most frequently demonstrated quality appraisal element, with 12 papers demonstrating the research they reported on was undertaken in response to a community-determined need or priority. The most frequently omitted quality appraisal elements related to how cultural and intellectual property rights were respected with no papers stipulating whether agreements were negotiated in regard to access rights to existing Aboriginal and Torres Strait Islander intellectual and cultural property or to protect ownership of intellectual and cultural property created through the research. 

## 4. Discussion

This scoping review considered the dietary assessment methods that have been used with Aboriginal and Torres Strait Islander people and communities and their applicability for use. The majority of studies took place in rural/remote settings, with only one study from an urban setting and one at the national population level. The analysis of store data was the most used dietary assessment method and only in rural/remote settings. This method seemed to be accepted by communities and was relatively efficient, inexpensive, and non-invasive. Weighed food records and FFQ had low applicability with issues relating to low cultural acceptance and questionnaires requiring understanding of concepts like ‘time’, ‘frequency’ and ‘quantity’. Modifications of conventional methods were commonly used to adapt to Indigenous practices, but only few studies incorporated Indigenous research methodologies such as yarning.

The finding that limited studies occurred in urban settings, compared to rural/remote settings, confirms similar findings by Whalan et al. in 2017 [14]. This does not accurately reflect the setting in which the majority of Aboriginal and Torres Strait Islander people and communities reside. With the exception of the NT, where just under 25% of Aboriginal and Torres Strait Islander people live in the Darwin area, at least one third of Aboriginal and Torres Strait Islander people in every other state and territory reside in urban rather than rural/remote areas [50]. A geographic disparity in study locations was also identified. The NT was overrepresented despite a comparatively small percentage (8%) of individuals who identify as Aboriginal and Torres Strait Islander living there [50]. In comparison, NSW, with only two studies [42,43], and QLD with one study [47], collectively represent over 60% of individuals who identify as Aboriginal and Torres Strait Islander [50]. The applicability of the dietary assessment methods may not translate across the distinctive cultures, knowledges, and experiences of Aboriginal and Torres Strait Islander people and communities from different urban, rural, and remote locations [4]. Representation and consideration of these differences through diversity of study locations and settings is essential. These findings indicate a need for evaluation of dietary assessment methods for their applicability with Aboriginal and Torres Strait Islander people and communities in a greater number of urban settings and geographic locations.

Low levels of cultural acceptance of the use or purpose of quantitative measures of dietary intake, particularly WFRs, were reported [41]. No strengths for the use of a WFR were identified in any of the four studies that utilised the method [41,42,43,44] and the limited completion of WFRs for more than one day was linked to incompatibility with the timing of traditional ceremonies [41]. Since the colonisation of Australia began, western forms of data collection on Aboriginal and Torres Strait Islander populations were used as evidence for monitoring, surveillance, interventions, and control by the state [51]. In the late 1900s there was a shift in social positioning where Aboriginal and Torres Strait Islander intellects challenged western hegemony towards privileging Aboriginal and Torres Strait Islander ways of ‘knowing, being, and doing’ in research principles. Moreover, assessed within the context of an Aboriginal and Torres Strait Islander understanding of ‘health’, a WFR may be considered less useful than in a western, biomedical context. ‘Health’, from an Aboriginal and Torres Strait Islander perspective, considers not only an individual’s physical health but also that individual’s, and their community’s, social, cultural, and spiritual wellbeing [3,4]. It is not possible to consider these elements using quantitative measures of individual intake, such as WFR. Dietary assessment methods that consider community-level intake or which allow for the collection of both qualitative and quantitative data through a combination of methods may better incorporate contextual elements (family life, shared meals, household infrastructure, and social and cultural events) of importance from an Aboriginal and Torres Strait Islander perspective.

This review identified conversational methods, with references to a yarning style approach, as a dietary assessment method used with Aboriginal and Torres Strait Islander populations [46]. While less recognised by a western scientific approach, yarning is a well-established Indigenous research method that facilitates the culturally safe sharing of information and knowledge at the discretion of participants [15,52]. While yarning may take many forms, ‘research yarning’ may occur with a semi-structured conversation-approached interview that seeks to gather knowledge and information from participants on a topic [15,52]. The way the conversational method was utilised by Bryce et al. allowed for contextualisation through the interweaving of a quantitative dietary assessment method, a modified 24-h recall (unstructured, minimal questioning or probing and no portion size estimation), and the food-related conversations had with household members [46]. Conversations from food and drinks consumed the previous day showed that ‘hungry days’ were very common, with differing food intakes on the first day of data collection compared to when a household member received income [46]. The conventional 24-h recall typically follows five steps (although some steps may be combined). Step 1: quick list (uninterrupted); Step 2: forgotten food list (series of food category questions); Step 3: time and occasion; Step 4: detail cycle (description, amounts, additions, review); Step 5: final review probe. This direct line of probing questions may not be a suitable approach to gather information amongst Aboriginal and Torres Strait Islander peoples. Furthermore, it may be more important to measure ‘usual intake’, rather than intake on one or a few days, considering the social impacts that may change eating patterns. Therefore, methods such as WFR and the 24-h recall may not be appropriate. The approach by Bryce et al. demonstrates a way in which both Indigenous and western research methodologies may be used together to collect quantitative and qualitative data to assess dietary intake more appropriately. Further investigation to validate the accuracy of methods that incorporate yarning-based approaches, or other Indigenous research methodologies, into quantitative data collection is needed. The importance of developing validated dietary assessment methods that are appropriate for this population cannot be understated considering the high susceptibility to nutrition-related health conditions such as malnutrition, overweight or obesity, diabetes, and cardiovascular disease.

A retrospective assessment of the cultural appropriateness of the peer-reviewed papers identified in this review was undertaken. There was no apparent trend in the quality rating, the use of a particular dietary assessment method, or the number of methods used. For example, papers rated ‘high’ included store data, FFQ, and mixed methods, papers rated ‘moderate’ used store data or mixed methods, and papers rated ‘low’ included store data, FFQ, WFR, observation, and mixed methods. The variation in quality score, despite the use of similar dietary assessment methods, indicates the need to consider the cultural appropriateness of the entire research process. Where this approach is taken, it is more likely the dietary assessment method will be selected in conjunction with, or approved by, community representatives and will gather data relevant to the nutrition-related priority identified by the community. Consideration of overall cultural appropriateness should also facilitate recognition of how diet and food make up just one element of a multifactorial concept of health, resulting in the understanding that a mixture of methods to capture relevant information may be needed [23]. 

The QAT findings also identified poor performance in all studies with respect to their reporting on ownership and control over existing cultural and intellectual property and such property created through research. The use of dietary assessment methods, gathering of related data, and reporting on it without clear agreements stipulating the use, control, and ownership of this information may be seen as a threat to Indigenous data sovereignty (being the right to autonomy over data collected, including how it is used, accessed, managed, interpreted, and re-used [2]). These results, in conjunction with the limited acceptance of certain quantitative dietary assessment methods discussed above, suggest there is a need for future studies collecting, analysing, and reporting on dietary intake to take, and report on, overt steps to create clear agreements regarding use and ownership of intellectual property and research materials. Such an approach may facilitate a shift from a datacentric western research approach towards a collaborative, strengths-based approach allowing self-determination by Aboriginal and Torres Strait Islander communities over data collected during the research process [2], including during the selection of culturally appropriate dietary assessment methods.

A strength of this review is the broad range of sources, including databases and grey literature, from which results were sought. Additionally, the lack of time limits on included results provided an expansive array of results and allowed for consideration of different approaches to dietary assessment and reporting styles over time. A limitation was the exclusion of specific Aboriginal and Torres Strait Islander population groups such as pregnant women, infants, and children, as well as studies that used only short dietary assessment instruments such as screeners. There is scope for future consideration of the applicability of short dietary assessment instruments or the dietary assessment methods that have been used with such specific Aboriginal and Torres Strait Islander population groups. Additionally, the cultural appropriateness assessment occurred retrospectively with the majority of papers published prior to the development of the QAT. It is recognised that papers published prior to the QAT’s development did not have the opportunity to consider their research or reporting against the QAT elements. The low scores should therefore not be viewed as discrediting previous research using dietary assessment methods with Aboriginal and Torres Strait Islander people and communities, but rather as an opportunity to identify areas for future research to continue to work to incorporate both Aboriginal and Torres Strait Islander and western, scientific approaches and priorities.

## 5. Conclusions

This review identifies the need for the prioritisation of cultural and contexualisation of social considerations of Aboriginal and Torres Strait Islander people and communities to guide the selection, use, and applicability of dietary assessment methods. Modifications of conventional methods were commonly used to adapt to Indigenous practices, but few studies incorporated Indigenous research methodologies such as yarning, which provides contextulisation of dietary intake. This highlights an opportunity for further investigation to validate the accuracy of methods that incorporate qualitative yarning-based approaches, or other Indigenous research methodologies, into quantitative data collection. The importance of developing validated dietary assessment methods that are appropriate for this population cannot be understated considering the high susceptibility to nutrition-related health conditions such as malnutrition, overweight or obesity, diabetes, and cardiovascular disease. This review also highlights the need for future research to focus on the applicability of dietary assessment methods in urban settings.

## Figures and Tables

**Table 1:** Summary of the study characteristics of included studies of the applicability of dietary assessment methods in Aboriginal and Torres Strait Islander Australians.

Dietary Assessment Method	Study Design	Population	Aims *	Dietary Components Assessed	Location	Study Name (If Applicable)	Reference
Store data	Cross-sectional	Community Rural/remote (*n* = 1700)	Explore the relationship between dietary quality and energy density of foods (MJ/kg) and energy cost ($/MJ) for an Aboriginal population living in a remote region	Contribution of food groups to dietary energy and dietary cost	Island community, Northern Australia	N/A	Brimblecombe et al., 2009 [26]
Store data	Cross-sectional	Community Rural/remote (*n* = 185–880; across 6 communities)	To examine the feasibility of using point-of-sale data to assess dietary quality of food sales in remote stores	1- nutrient profiles (macronutrient contribution to energy and nutrient density—nutrient/1000 kJ); 2- major food sources of macro- and micronutrients	Remote Australia, three states & NT	Remote Indigenous Stores and Takeaway Project (RIST)	Brimblecombe et al., 2013 [27]
Store data	Stepped wedge randomised controlled trial	Community Rural/remote (*n* = 8515; 20 communities)	To measure the effect of a price discount on food and drink purchases with and without an instore consumer education strategy applied at the population level	Population intake and estimated intake/capita. Purchases in grams of fruit and vegetables, water, artificially sweetened soft drinks, SSB, healthy food, discretionary food, other beverages, and Australian Health Survey food groups and nutrients	NT (20 remote Indigenous communities)	Store Healthy Options Project in Remote Indigenous Communities (SHOP@RIC)	Brimblecombe et al., 2017 [28]
Store data	Multi-site 12-month audit	Community Rural/remote (*n* = 2644; 3 communities)	To describe the nutritional quality of community-level diets in remote northern Australian communities	Community-level dietary intake (energy, food type, quantity, micronutrients and macronutrients, and food sources of nutrients)	NT (3 remote communities)	N/A	Brimblecombe et al., 2013 [29]
Store data	Non-randomised trial with baseline as control	Community Rural/remote (*n* = 154 (mean))	Summarise the development and testing of the store-turnover method, a non-invasive dietary survey methodology in remote, centralised Aboriginal communities	Changes in nutrient density over intervention period and changes in apparent consumption of targeted foods (e.g., fruit and vegetables, wholemeal bread, white sugar)	NT (Minjilang, Croker Island)	Minjilang Health and Nutrition Project	Lee 1993 [31]
Store data	Non-randomised trial with baseline as control	Community Rural/remote	To describe an unusually successful health and nutrition project initiated by the people of Minjilang	Apparent community dietary intake and change—changes in nutrient density and apparent consumption compared to biological markers	NT (Minjilang, Croker Island)	Minjilang Health and Nutrition Project	Lee et al., 1994 [32]
Store data	Non-randomised trial with baseline as control	Community Rural/remote	Assess the long-term effect of a nutrition program in a remote Aboriginal community and report the nutritional outcomes up to 1993 after monitoring ceased in June 1990	Changes in dietary intake of target foods (including fruit, vegetables, and wholegrain bread) and nutrients (including folate, ascorbic acid, and thiamine)	NT (Minjilang, Croker Island)	Minjilang Health and Nutrition Project	Lee et al., 1995 [33]
Store data	Cross-sectional	Community Rural/remote (*n* = 1617; 6 communities)	Describe the apparent per capita food and nutrient intake in six remote Australian Aboriginal communities	Mean daily dietary intake per capita. Contribution of macronutrients to total energy	NT (6 remote communities—3 central desert, 3 northern coastal)	N/A	Lee et al., 1994 [30]
Store data	Cross sec-tional	Community Rural/remote (total population not specified)	To document change in prevalence of obesity, diabetes, CVD risk factors, and trends in dietary macronutrient intake over an eight-year period in a rural Aboriginal community	General trends in food consumption expressed as nutrient density (sugar, total fat, saturated fat, complex carbohydrates as percentage of total energy)	NT (Rural community)	N/A	McDermott et al., 2000 [34]
Store data	Pre and post survey	Community Rural/remote (total population not specified)	Evaluate the effectiveness of a community-directed intervention program to reduce coronary heart disease risk through dietary modification	Changes in dietary quality	WA (Kimberley region)	Looma Healthy Lifestyle Program	Rowley et al., 2001 [35]
Store data	Cross-sectional and retrospective comparision	Community Rural/remote (*n* = 1646; 5 communities)	To provide feedback to relevant Councils to help inform decision making on key issues and to identify changes in food supply 1986–2012	Energy, macronutrient, and micronutrient data. Results compared with dietary recommendations from ADGs and reported separately for each store	SA (Anangu Pitjantjatjara Yankunytjatjara (APY) Lands)	N/A	Lee et al., 2013 [49]
FFQ	Cross-sectional	Individual Rural/remote (*n* = 135)	To assess prevalence of modifiable health-risk behaviours among Indigenous Australian adults with diabetes	Food group intake including: vegetables, fruit, fish, takeaway meals, liquids (water, milk, juice, diet and regular soft drink), snacks	VIC (Mooroopna)	Diabetes Education and Eye Screening (iDEES) project	Atkinson -Briggs et al., 2022 [36]
FFQ	Cross-sectional study	Individual Rural/remote (*n* = 209)	To describe health behaviours of Indigenous Australians with diabetes attending a primary care clinic	Frequency of food group intake of: vegetables, fruit, fish, takeaway meals, liquids (water, milk, juice, diet and regular soft drink), snacks	NT (Remote community clinic, Alice Springs)	TEAMsnet project	Xu et al., 2019 [37]
FFQ (Modified “food list” and questionnaire method)	Cross-sectional	Community Rural/remote (17 settlements; population range: 23–300)	Evaluate the dietary intake, food consumption patterns, and eating habits of Aborigines* living on government settlements, missions, and cattle stations	Total food intake per week (meat, sugar, tea, flour, cereal and bread, fruits and vegetables). Intake of specific nutrients (vitamin C, calcium, vitamin A)	NT (Darwin, Gulf of Carpentaria, Alice Springs, Barkly Tableland)	N/A	Wilson 1953 [38]
RAP- modified FFQ Interviewing ‘key’ informants Focus groups	Cross-sectonal	Community Rural/remote (*n* = 25)	Review the published qualitative data using RAP to describe distant past food intake on cattle stations prior to the 1960s and food intake of Aborigines* aged 50 and over in 1988 in Junjuwa	Contribution of individual foods consumed to diet quality	WA (Junjuwa, Fitzroy Crossing)	‘Food Habits in Later Life’ (FHILL) Program 1988–1993	Kouris-Blazos & Wahlqvist 2000 [39]
RAP-modified FFQ Weighing of food Observation Group discussions	Cross-sectional	Community Rural/remote (*n* = 15)	Ascertain the major nutritional problems in an Aboriginal Australian elderly community, document the risk factors, especially dietary, that may be contributing to the deterioration in health	Average daily intake of energy, protein, fat, carbohydrates. Food habits, including quality and quantity of foods consumed on “binge” days versus “lean” days	WA (Junjuwa)	‘Food Habits in Later Life’ (FHILL) Program 1988–1993	Wahlqvist et al., 1991 [40]
WFR 24-h recall FFQ Diet history Store data	Cross-sectional comparision	Community Rural/remote (coastal *n* = 302; desert *n* = 247; weighed and recalled intake *n* = 41)	Identify a practical quantitative dietary survey methodology acceptable to remote Aboriginal communities, to assess the face validity of each method and to compare quantitative data obtained	Macro and micronutrients, percent energy derived from protein, total carbohydrate, fat, sugars, complex carbohydrate, apparent consumption of food groups	Northern coastal community and central desert community (anonymous)	N/A	Lee et al., 1995 [41]
WFR 6-day Observation Questioning	Cross-sectional	Community Rural/remote (*n* = 720)	Reports the extent of vitamin deficiency in a community of part-Aboriginal* community	Foods available and consumed and micronutrients content. Weighed food record used to confirm community level dietary observations	NSW (Bourke)	N/A	Kamien 1974 [42]
WFR 6-day (Mon-Sat)	Cross-sectional	Individual Rural/remote (*n* = 17)	A detailed investigation of diet and nutrition of two Aboriginal families, to assess prevalence the extent of biochemical and clinical nutrition deficiency found amongst the Aboriginal population of Bourke	Food groups, intake and percent contribution to calories, protein and micronutrients	NSW (Bourke)	N/A	Kamien 1975 [43]
WFR 28-day	Cross-sectional	Individual Rural/remote (*n* = 23)	Investigate possibility that dietary factors other than vitamin A, influence plasma retinol and beta-carotene level in apparently healthy adult individuals consuming their usual diet	Energy, macronutrients, retinol, beta-carotene and total vitamin A, dietary fibre and zinc	WA (Fitzroy Crossing)	N/A	Rabuco et al., 1991 [44]
Observation	Cross-sectional	Community Rural/remote (total population not specified)	Provide a general report of food consumption and dietary levels of the Aborigines* at the settlements. Biochemical tests to assist in assessment of nutritional status	Calories, protein, iron, calcium, specific vitamins	NT (Four settlements in Arnhem Land)	American-Australian Scientific Expedition to Arnhem Land	McArthur et al., 2000 [45]
Observation Conversational methods 24-h recall	Cross-sectional	Household Rural/remote (13 households with 4–13 occupants)	1. What food and drinks Anangu families are eating; and 2. Factors that influence these food choices	Food and drinks consumed, foods and drinks purchased. Collection of receipts. Records of food preparation: how, when and who was eating. Food groups as defined in the ADGs, and energy and macronutrient content	SA (Anangu Pitjantjatjara Yankunytjatjara (APY) Lands)	Maitjara Wangkanyi (“talking about food”)	Bryce et al., 2020 [46]
Observation Adapted store data Adapted 24-h recall	Cross-sectional	Community Rural/remote (*n* = 69)	An anthropological study to provide information on the diet and lifestyle of the Aboriginal owners of Maralinga and Emu to determine possible options for the future rehabilitation of the Maralinga lands	Average consumption in grams per capita per day for a range of bush foods and store foods	SA (Oak Valley, Maralinga Lands)	N/A	Palmer & Brady 1991 (2nd ed. 2021) [48]
Multiple-pass 24-h recall (baseline and 12 months) and casual ‘yarn’	Prospective cohort	Community Urban (*n* = 100)	Evaluate reported change in nutrient intake of adult urban Aboriginal and Torres Strait Islander people who participated in a medically based lifestyle intervention program	Nutrient intake and food group serves	QLD (Townsville)	Walk-about Together Program (WAT)	Longstreet et al., 2008 [47]
24-h recall (2 at least 8 days apart in non-remote areas and one in remote areas)	Cross-sectional survey	National Rural/remote /urban (*n* = 4109)	Collect detailed nutrition information from Aboriginal and Torres Strait Islander people	Food groups, energy, and nutrient intakes	Australia	National Aboriginal and Torres Strait Islander Nutrition and Physical Activity Survey 2012 -2013 (NATSINPAS)	Australian Bureau of Statistics, 2015 [6]

FFQ = food frequency questionnaire, WFR = weighed food record, RAP = rapid assessment procedures, CVD = cardiovascular disease, SSB = sugar sweetened beverage, ADGs = Australian Dietary Guidelines, MJ = megajoule, kJ = kilojoule, kg = kilogram, NSW = New South Wales, VIC = Victoria, QLD = Queensland, WA = Western Australia, NT = Northern Territory, SA = South Australia, N/A = not applicable. * language used in aims comes directly from the original paper.

**Table 2:** Reported strengths and limitations of identified dietary assessment methods to understand the applicability for Aboriginal and Torres Strait Islander people and communities by location.

Methods Identified in Review	Reported Strengths	Reported Limitations
URBAN POPULATIONS		
24-h recall [47]	35% rate of underreporting identified in the study compares to underreporting identified in other studies, suggesting appropriateness of method [47]. Strategies used identified as helpful by participants similar to those used with other populations [47].	Underreporting likely attributable to social desirability of lower intake [47]. Weekend intake missed if recalls cover weekdays only [47]. Intake of bush foods may be underreported if more often consumed on weekends and recall focuses on weekdays [47].
RURAL POPULATIONS		
FFQ [36,37,38,39,40,41]	Questionnaire adapted to include checklist of geographically specific bush foods [39,40]. Portion size estimates able to be assisted with photographs, food scales, play dough, and items from supermarket [40].	Self-reporting may lead to bias towards perceived correct answers e.g., underreporting discretionary foods [36,37]. Unreliable recollection [36]. Converting intake frequency using standard serving sizes may underestimate serves if actual serving sizes larger than standard [37]. Modified questionnaire likely insensitive to gender difference in intake [39]. Issues with questionnaire requiring understanding of concepts of ‘time’, ‘frequency’, and ‘quantity’ [39,40]. Produced list of foods eaten at some point or enjoyed not actual intake [41].
Modified food list/ questionnaire [38]	None identified.	Data based on food amounts issued rather than consumed [38]. Risk of no allowance for seasonal variations [38]. Uneven distribution of food and wastage not accounted for [38]. No consideration of bush foods consumed [38].
WFR [41,42,43,44]	None identified.	Presence of strangers at meals may change eating habits [43]—weighing of food carried out by survey team who were present during meal preparation and at meal times. Participants anxious to present a good image [43]. Timing of assessment during periods of employment and pension week resulting in greater food purchases than usual [43]. Reported non-compliance during recording of food [44]. Poor compliance for record greater than one day, linked to timing of traditional ceremonies and financial difficulties [41]. Close supervision provided (at least 3 times per week, often daily) to facilitate compliance [44]. Difficultly ensuring all foods consumed were weighed and observed [41]. Low cultural acceptance of usefulness of quantitative measures of intake [41].
Diet history [41]	None identified.	Difficulties stating ‘usual’ intake may be related to significant day-to-day variability in diet [41].
24-h recall [41,46,48]	Minimal questioning or use of probes during recall and without attempts to estimate portion sizes * [46]. May be adapted to best suit circumstances such as visiting participants twice per day (every 12 h) and asking about foods eaten in the last 12 rather than 24 h [48]. Helped confirm frequency of ‘hungry’ days (i.e., days where two or more meals missed or minimal food consumed) [46].	Foods frequently underestimated (sugar, sweetened beverage) and others overestimated (fruit, veg, and traditional foods) [41]. Evidence of selective recall and bias in responses may be related with tendency to ‘please’ interviewer [41]. Difficulties repeating method with same participants due to frequent population movement [48].
Observation/conversational methods [42,45,46,48]	Considers factors influencing food choices (e.g., food shopping and cooking observed) [46,48]. Allows inclusion of difficult to quantify foods or foods that may be omitted from store sales data such as bush foods and foods entering community through other avenues e.g., visitors [48]. Allows contextual dietary information to be collected (e.g., income, number of household members/visitors who ate foods, cooking facilities, number of ‘hungry’ days, eating patterns) [46]. Greater control over disclosure for participants [46].	Inability to accurately quantify and describe food gathered particularly on weekends [45]. Prescence of observers influences foods procured and consumed [48]. Reliance on method may contribute to underestimation of consumption of bush foods and foods bought by visitors [48].
Focus groups/ group discussions [39,40]	Reveals food quality and quantity of consumption closely connected to weekly pension [39]. RAP (open ended question approach) allowed questions on health and lifestyle as well as food intake [40].	None identified.
Store data [26,27,28,29,30,31,32,33,34,35,41,48,49]	Assess dietary quality of food sales in remote community stores, trends in intake between communities [27]. Objective proxy for community-level dietary intake where community stores provide main source of food [27,28,30,31,32,41]. Specific to relatively small geographical areas [41]. Efficient and inexpensive [30,31,32,41]. Relatively non-invasive [30,32,41]. Higher acceptability to communities compared to individual dietary intake methods [41]. Less potential for bias as no reliance on subjective assessment of intake and avoids language, literacy, and numeracy factors that may reduce reliability of direct measurement or recall [30,41]. Allows for retrospective data to be collected [41]. Seasonal variation in intake may be determined [32]. Allows for within-store comparison, e.g., changes in purchasing data [28]. Can identify contributions of specific food and beverage items for targeted community-based intervention strategies and policy [27,30]. Can be used to evaluate impact of community-based nutrition programs [30,31,32]. Can be utilised by Aboriginal researchers and health workers within communities for increased community involvement in health research and promotion [41]. Gathered nutrition information may be easily relayed to communities in culturally specific ways [30].	Provides estimate of food and nutrient availability, not actual intake [27]. Usefulness dependant on contribution of store to overall population-level diet [27]. Large variation in fortnightly sales [28]. Insensitive to food preparation and cooking methods [27,35]. Does not account for wastage after purchase [27,30,48]. Does not account for food/beverages obtained outside store, e.g., bush food [26,30,35,49]. Estimates of community-level energy/nutrient intakes rely upon accurate population estimates, problematic where population mobility high [27,29,30,41,48]. Cannot assess food distribution patterns within the community [30,41,48]. Cannot identify individual or sub-group dietary intake or diet composition [30]. Estimated dietary quality limited by accuracy of food composition databases, e.g., nutrient composition of perishable items may be impacted by long-distance transportation [27,30]. Errors in measurement in method such as missing invoices, stock with very slow turnover [30]. Not adequate for nutritional surveillance of community [41].
NATIONAL DATA		
24-h recall [6]	To account for seasonal variations in nutrition may be conducted over 12-month period [6]. Trained interviewers can conduct interviews [6]. Can be conducted in non-remote and remote areas [6].	Underreporting and social desirability bias, especially with discretionary food intake [6]. Systematic underreporting risk with children unable to recall intakes and carers may be unaware of total intake of child [6]. Difficulties comparing results with guidelines and risk of overestimation of proportion meeting recommendations if units different to guidelines (e.g., whole serves where guidelines for some sex/age groups are half serves) [6]. Reliability may be influenced by sampling error [6]. Risk of undercoverage (i.e., difference between population represented by the sample size of the survey and the in scope population) [6]. Difficulty conducting second recall in remote areas [6] Accuracy of recall data dependent on accuracy of measures, food databases [6].

FFQ = food frequency questionnaire, WFR = weighed food record, RAP = rapid assessment procedures. * Categorised as a strength of the method due to the ability to modify the application of the method to fit within a conversational approach consistent with cultural norms and the sharing of knowledge through yarning [46].

## Data Availability

Not applicable.

## Supplementary Materials

The following supporting information can be downloaded at: https://www.mdpi.com/article/10.3390/nu15030787/s1, Table S1: Medline search strategy; Table S2: 2018 SAHMRI CREATE Aboriginal and Torres Strait Islander Quality Appraisal Tool: results of peer-reviewed papers; Table S3: Brief summary table on the main features contained in each dietary assessment method.
