# Supplementary material for: Yarning about Diet: The Applicability of Dietary Assessment Methods in Aboriginal and Torres Strait Islander Australians—A Scoping Review
Source: Nutrients. 2023 Feb 3;15(3):787. doi: 10.3390/nu15030787 (PMC9919225; doi:10.3390/nu15030787)
Supplement: Supplementary file 1 [file nutrients-15-00787-s001.zip › nutrients-2073403-supplementary.pdf]

Supplementary Table S1. Medline search strategy.

Search	Query	Records retrieved
**1**	Indigenous Peoples/<br>"Native Hawaiian or Other Pacific Islander"/<br>Health Services, Indigenous/<br>(Aborigin* OR Indigen* OR First People* OR First Nation* OR 1st nation* OR Native OR Korri OR Murri OR Nyoongah OR Goori OR Yolngu OR Anangu OR Palawa OR Ngarrindjeri OR Torres Strait Island* OR Murry Island People* OR Mer Island People*).tw	296,702
**2**	exp Australia/<br>(Austral* OR Northern Territor* OR Victoria* OR Queensland* OR New South Wales OR Tasmania OR NSW OR NT OR WA OR VIC OR SA).tw<br>(Austral* OR Northern Territor* OR Victoria* OR Queensland* OR New South Wales OR Tasmania).in	985,471
**3**	Exp Nutrition Assessment/ OR nutritional status/ OR Nutrition Surveys/ OR Diet Surveys/ OR Diet Records/ OR Energy Intake/ OR<br>Artificially Sweetened Beverages/ OR Sugar-Sweetened Beverages/ OR Alcoholic Beverages/ OR Carbonated Beverages/ OR Beverages/ OR Fermented Beverages/<br>OR (store adj3 (survey* OR rural OR outback OR sale* OR data OR regional OR remote OR turnover)).tw<br>OR (nutri* adj3 (survey* OR intake* OR app* OR assess* OR health OR intervention*)).tw<br>OR (diet* adj3 (pattern* OR intake* OR assess* OR survey* OR record* OR data OR intervention* OR recall* OR method* OR 24-h* OR image* OR app* OR estimat*)).tw<br>OR (food adj3 (questionnaire* OR intake* OR survey* OR consum* OR estimat* OR weigh* OR record* OR recall*)).tw<br>OR nutrit*.tw<br>OR (tucker OR grub).tw	551,461
**4**	1 AND 2 AND 3	756

Supplementary Table S2. 2018 SAHMRI CREATE Aboriginal and Torres Strait Islander Quality Appraisal Tool: Results of peer-reviewed papers.

**First Author, Reference**	**Indigenous Governance**				**Respect for cultural and Intellectual Property**				**Capacity Building**			**Beneficial Outcomes**			**Overall Assessment**
	**Q1**	**Q2**	**Q3**	**Q4**	**Q5**	**Q6**	**Q7**	**Q8**	**Q9**	**Q10**	**Q11**	**Q12**	**Q13**	**Q14**	
**Atkinson - Briggs et al.**	Y	Y	N	N	Y	N	N	Y	Y	Y	Y	Y	Y	P	High
**Brimblecombe et al. 2009 [26]**	Y	Y	N	N	N	N	N	P	N	N	N	N	N	N	Low
**Brimblecombe et al. 2013 [27]**	Y	N	N	N	N	N	N	N	N	N	P	N	N	N	Low
**Brimblecombe et al. 2013 [29]**	Y	N	N	N	N	N	N	N	N	N	N	N	N	N	Low
**Brimblecombe et al. 2017 [28]**	Y	Y	Y	Y	Y	N	N	Y	N	N	Y	Y	Y	Y	High
**Bryce et al. 2020 [46]**	Y	Y	Y	Y	Y	N	N	Y	Y	Y	U	Y	Y	Y	High
**Kamien 1974 [42]**	N	N	N	N	N	N	N	N	N	N	N	P	N	N	Low
**Kamien 1975 [43]**	N	N	N	N	N	N	N	N	N	N	P	Y	P	P	Low

First Author, Reference	Indigenous Governance				Respect for cultural and Intellectual Property				Capacity Building			Beneficial Outcomes			Overall Assessment
**Kouris-Blazos & Wahlqvist 2000 [39]**	N	P	N	N	N	N	N	N	N	N	P	N	N	N	Low
**Lee 1993 [31]**	Y	Y	Y	Y	Y	N	N	Y	Y	Y	Y	Y	Y	Y	High
**Lee et al. 1994 [32]**	Y	Y	Y	Y	Y	N	N	Y	Y	Y	Y	Y	Y	Y	High
**Lee et al. 1994 [30]**	N	P	N	N	N	N	N	P	N	N	P	P	N	N	Low
**Lee et al. 1995 [33]**	Y	Y	Y	Y	Y	N	N	Y	Y	Y	Y	Y	N	Y	High
**Lee et al. 1995 [41]**	N	Y	N	N	Y	N	N	N	N	N	N	N	Y	P	Low
**Longstreet et al. 2008 [47]**	Y	N	N	N	N	N	N	N	Y	P	Y	Y	Y	Y	Moderate
**McArthur et al. 2000 [45]**	N	N	N	N	N	N	N	N	N	N	N	N	N	N	Low
**McDermott et al. 2000 [34]**	P	Y	N	N	N	N	N	N	N	N	N	Y	Y	Y	Low
**Rabuco et al. 1991 [44]**	N	N	N	N	N	N	N	N	N	N	N	N	N	N	Low
**Rowley et al. 2001 [35]**	Y	Y	U	U	N	N	N	Y	N	N	P	Y	Y	P	Moderate

First Author, Reference	Indigenous Governance				Respect for cultural and Intellectual Property				Capacity Building			Beneficial Outcomes			Overall Assessment
**Wahlqvist et al. 1991 [40]**	N	N	N	N	N	N	N	N	N	N	N	N	N	N	Low
**Wilson 1953 [38]**	N	N	N	N	N	N	N	N	N	N	N	N	N	N	Low
**Xu et al. 2019[37]**	N	N	N	N	N	N	N	N	N	N	N	N	N	N	Low

Y = Yes, P = Partially, U = Unclear, N = No explicit statements in the body of the text to provide evidence for each question below

1. Did the research respond to a need or priority determined by the community?
2. Was community consultation and engagement appropriately inclusive?
3. Did the research have Aboriginal and Torres Strait Islander research leadership?
4. Did the research have Aboriginal and Torres Strait Islander governance?
5. Were local community protocols respected and followed?
6. Did the researchers negotiate agreements in regards to rights of access to existing Aboriginal and Torres Strait Islander peoples' intellectual and cultural property?
7. Did the researchers negotiate agreements to protect Aboriginal and Torres Strait Islander peoples' ownership of intellectual and cultural property created through the research?
8. Did Aboriginal and Torres Strait Islander peoples and communities have control over the collection and management of research materials?
9. Was the research guided by an Indigenous research paradigm?
10. Does the research take a strengths-based approach, acknowledging and moving beyond practices that have harmed Aboriginal and Torres Strait peoples in the past?
11. Did the researchers plan to and translate the findings into sustainable changes in policy and/or practice?

Table S3. Brief summary table on the main features contained in each dietary assessment method.

**Dietary Assessment Methods**	**Description**
Store Data	Either store sales/receipt data or store turnover from the analysis of invoices of foods supplied to stores.
24-hour recall	Provides information on the respondents exact food and beverage intake (types and quantities) during a 24-hour period. The elements of the 24-hour recall typically include step 1: quick list (uninterrupted); step 2: forgotten food list (series of food cat. questions); step 3; time and occasion; step 4: detail cycle (description, amounts, additions, review); step 5: final review probe. Some steps may be combined.
Food frequency questionnaire	Usual intake estimates over a relatively long period (e.g. past 6 months, past year). Usual frequency of consumption of each food from a list of foods, using a set of frequency-of-use response categories. A reference amount of food may be provided to estimate quantities.
Weighed food record	Detailed prospective dietary assessment method. All details of foods/drinks consumed are reported at the time of consumption. Instructions and record booklets (or apps) and a set of weighing scales are provided. Portions of food are weighed and described in detail.
Diet history	Usual intake estimates over a relatively long period (e.g. past month, past 3 months, past year). Typically includes 1) an in-depth interview to determine usual meal patterns, and 2) a food checklist to cross-check information obtained in part 1. Need both frequency of consumption of the foods and portion size.
Direct observation	Observation of dietary intake and food practices by research staff. Food intake is visually estimated or measured.
Conversational methods	Include the use of focus groups, group discussions and yarning. Focus groups and group discussions collect qualitative dietary data such as nutrition knowledge, attitudes, and intake patterns. Yarning is a conversational, informal interview through which participant and interviewer journey together to gather information during the interview process.
